# Exploring loneliness in elderly Javanese and social support

**DOI:** 10.3389/fsoc.2025.1528886

**Published:** 2025-09-01

**Authors:** Ayu Diah Amalia, Achmadi Jayaputra, Nurasih Shamadiyah

**Affiliations:** ^1^Indonesian Social Affairs Ministry, Jakarta, Indonesia; ^2^National Research and Innovation Agency, Jakarta, Indonesia; ^3^Department of Agribusiness, Malikussaleh University, North Aceh, Indonesia

**Keywords:** elderly loneliness, social support, culture, wellbeing, mental health

## Abstract

Loneliness is a common issue among the elderly, often resulting from reduced social interactions which negatively impact their mental and physical health and overall wellbeing. This study aims to explore the experience of loneliness in elderly individuals and examine how cultural factors and social support can mitigate this condition. Eleven women aged 60 and older, living alone in Simomulyo Baru Village, Surabaya, Indonesia, participated in the study. Data were collected via in-depth interviews and observations, and analyzed using a narrative analysis approach. Contrary to common assumptions, most participants did not report feeling lonely despite living alone. The collectivist culture known as Guyub, alongside strong social support, notably material, emotional, and companionship support, were identified as critical factors that reduce loneliness. However, instrumental and informational supports were less available. The findings highlight the substantial role of collectivism and social support, especially from family and community, in alleviating loneliness among the elderly. Supporting and strengthening family and community networks to create elderly-friendly environments is essential. Further research should focus on the influence of cultural and social contexts on elderly loneliness.

## 1 Introduction

Social changes have made the elderly vulnerable, manifesting in dependence on others, limited capacities, and reduced social connections (Langmann, 2023). These changes contribute to loneliness among elderly, primarily due to diminished intergenerational relationships, reduced mobility, and increasingly individualized communities (Fakoya et al., 2020; Zhao and Wu, 2022). Loneliness in elderly adversely affects both physical and mental health, potentially leading to depression (Isik et al., 2021), heightened anxiety, worsening cognitive and functional impairments, and increased mortality risk (Choi et al., 2022).

Loneliness is strongly linked to mental health (Cacioppo and Cacioppo, 2018), often described as an unpleasant condition where the actual number or quality of relationships falls short of expectations. It is a subjective, negative experience stemming from an individual's cognitive evaluation of the quantity and quality of their relationships (de Jong Gierveld et al., 2006). Loneliness can manifest as either social loneliness, where the number of social connections is insufficient, or emotional loneliness, where the desired closeness in relationships is not achieved (de Jong Gierveld and van Tilburg, 2010).

Loneliness is influenced by personal background, with lower levels typically observed in collectivist societies compared to individualist ones, although exceptions exist (Chow et al., 2021). Individuals experience loneliness differently, shaped by cultural and social factors. Loneliness is more prevalent in individualistic cultures (Barreto et al., 2021). Culture shapes how individuals form relationships and maintain social cohesion within society (Heu et al., 2021). In addition to culture, social support can mediate loneliness and depression in the elderly (Bareket-Bojmel et al., 2021; Kim and Lee, 2022; Liu et al., 2016). A smaller social support network among the elderly may increase feelings of loneliness (Chen et al., 2014; de Guzman et al., 2012). Elderly often experience loneliness due to the loss of familial and cultural bonds and their inability to participate in social activities because of physical, psychological, and social changes. They may struggle to form new friendships and integrate into new social networks (Singh and Misra, 2009). Loneliness is associated with a lack of social networks and reduced time spent in social activities (Nitin et al., 2016).

Social support helping to reduce loneliness in elderly (Elsayed et al., 2019; Sung et al., 2011). A robust social support system is essential, and elderly should be encouraged to engage in social activities (Arslantaş et al., 2015). Social support is crucial for alleviating loneliness in elderly, as the absence or loss of support systems can leave them vulnerable. When social support is limited, technology can be considered to help the elderly remain socially connected with family, friends, and the broader community (Melchiorre et al., 2024).

The lack of social relationships and family support can exacerbate loneliness in elderly, so their participation in social activities is vital to improving mental health (Manchana, 2024). Social relationships can be understood through the lens of social support and social networks. Social support refers to resources provided by others in times of need, namely the extent to which one can rely on their social network for help, care, and comfort (Santos and Hamdan, 2024). Well-designed social connection schemes play a crucial role in this process, ultimately safeguarding health and wellbeing (McDaid and Park, 2024).

The support provided to elderly by others constitutes a form of social support, typically offered by family, friends, neighbors, professionals, and social organizations or institutions. For the elderly, family members generally serve as the primary sources of support, including spouses, siblings, children, and other family relations. Sources of support categorized into three groups: close kin (spouses, children, parents, siblings), extended kin (uncles and aunts), non-kin primary group (friends, neighbors, coworkers), and secondary groups (professionals, volunteer agencies, and government departments) (Liu, 2019).

Support functions are divided into material support, emotional support, companionship support, instrumental support, and informational support. Material support refers to the provision of tangible resources such as money or goods that form the economic basis of life. Emotional support includes expressions of love, empathy, care, active listening, and trust, offering relief from negative emotions. For elderly, when faced with difficult problems or worries, they often seek psychological comfort from someone. Companionship support relates to the need for company, especially among elderly individuals who live apart from their children or live alone, driving them to seek companionship to alleviate loneliness. This type of support reflects the increasing need brought about by urbanization and industrialization. Instrumental support is defined as services, actions, and other non-material forms of assistance that directly help individuals in need. In addition to family members, social workers, doctors, and other professionals can provide instrumental support to the elderly. For elderly living in care facilities, they may receive instrumental support from caregivers. Informational support refers to the provision of useful information that can be applied to solve problems, enabling individuals to address issues independently. It serves as a medium for offering help and guidance, including knowledge and skills necessary to navigate various stressful situations. Moreover, advice, cognitive guidance, feedback, and directions are also part of informational support, as they aid in problem-solving. Today, the elderly are often encouraged to live independently through empowerment, where informational support plays a key role. Later life is perceived as a period with limited future time, leading to greater emotional needs. Emotional and companionship support becomes more meaningful to the elderly. In this era of an advanced information society, individuals can access a wide range of information. Elderly living in communities can receive information about matters of interest to them, including options for their social wellbeing (Liu, 2019).

Loneliness among the elderly can negatively impact their health and wellbeing. It is crucial to explore how the elderly interpret loneliness and how they live with and manage it (Barbosa Neves et al., 2019). The study by Sung et al. (2011) explained the relationship between social support and loneliness in elderly women who live alone, and that it has a good impact on the elderly. The study of Liu et al. (2016) revealed that social support for the elderly provides evidence that it can reduce depression among the elderly. Social support can be a potential protective factor for the lonely elderly. Emotional and social support play an essential role in protecting the elderly from depression. It is considered an intervention to reduce depression and improve the quality of life of the elderly. This study examines loneliness among participants and how culture and social support can alleviate loneliness among the elderly in Simomulyo Baru Village, Surabaya, East Java, the second-largest city in Indonesia. This study refers to the concept of social support put forward by Liu (2019) in identifying the availability of social support and cultural functions in the community to the elderly.

## 2 Design and method

This narrative analysis study explored the participants' experiences of loneliness and how culture and social support helped alleviate it by focusing on the participants' stories of loneliness, paying attention to how these stories were constructed, for whom, and why, as well as the cultural discourse underlying them. Narratives were used to organize and sequence their life experiences (Ntinda, 2018). Narrative research aims to understand individual experiences and their meanings (Varnaseri and Alhaei, 2022). Phenomenon analysis is a type of narrative analysis that focuses on the narrator's subjective experience, and how they interpret and make sense of their experience. Researchers analyzed the language used to describe experiences, emotional expressions, and the way in which they constructed the meaning of their experiences. Phenomenological analysis can be useful for understanding how they make sense of life and experiences (Hassan, 2024). The daily life and stories of the elderly are explored to get a comprehensive narrative picture of the meaning of loneliness. We also observe the social settings of participants, culture, and social supports in the community.

### 2.1 Participant

This study involved 11 elderly individuals aged 60 years and above, as defined by the Indonesian Elderly Law. The youngest participant was 60 years old and the oldest was 81 years old. They resided in Simomulyo Baru Village, Surabaya, Indonesia. Simomulyo Baru Village is a densely populated residential area located in the city center, surrounded by commercial and office districts. The area's high mobility and individualistic tendencies were characteristic of its population. Residents in this area are lower-middle class and upper-middle class. Elderly participants live in national housing with small, medium, and large house sizes. Their social class was identified by the type of house size, income (pension and income from work), education, and occupational background. Most participants did not receive social assistance, three elderly people had economic limitations, and only one participant received social assistance. Participants also did not spend money to participate in elderly activities, but were given social assistance from the local government, such as food for the elderly in elderly activities. Participants who participate in social activities in the local community incur costs (dues) for consumption, but the costs are very low. Nine elderly participants are migrants outside Surabaya city, but have lived in the residence for a long time. The criteria for participation included elderly women living alone. In this area, older women experienced solitude more frequently, as they generally did not remarry after losing their spouses and had a higher life expectancy compared to men. The search for participants is based on population administrative information from community leaders and elderly leaders. The researcher then selects participants based on the criteria of living alone and permanent residential status or having lived in the location for a long time (having lived for decades). This criterion is a consideration because they understand the social and cultural setting of the community. Triangulation was also conducted to improve data quality by interviewing several individuals, such as neighbors, the community, and elderly leaders.

### 2.2 Data collection

Data were collected through semi-structured interviews and observations. The researcher conducted interviews at the participants' homes for about 1–2 h. We also observe the participants' residential environment, and neighborhood environment, and at certain times see participants in daily life activities. The researcher also explored the life stories and characters of the participants to explore further the conditions of loneliness they experienced. In the cultural context, data collection is carried out by conducting interviews and observations of individual and community social practices. We also observed the participants' daily activities through their social media. On the theme of the context of social support, the researcher conducted interviews and explored the sources and types of social support that participants received.

### 2.3 Data analysis

Individual storytelling facilitated the social and cultural construction of identity. Structural analysis of the transcripts provided a more concrete storyline (Nasheeda et al., 2019). Researchers conduct narrative analysis focusing on themes, motifs, and other patterns through the coding process. Analyze the formal structure of the narrative, the use of language, and the cultural context in which the situation is addressed. Then interpret the findings in narrative analysis, describing conclusions regarding meaning, experience, and perspective (Hassan, 2024).

We transcribed the results of the interviews and conducted observation reports of participants and other parties involved in the study. Using NVivo software, we create codes on themes explored, such as loneliness, culture, and social support, and analyze them. Apart from that, we also identify the identity of the participants, such as age, gender, ethnicity, number of families, marital status, residential status, economic status, social status, and health status. All of the data is given a code to facilitate analysis and associate themes with subthemes, intended for researchers to delve deeper into the condition of participants and understand the loneliness conditions of participants.

## 3 Result

This section presents and discusses participants' experiences of loneliness, the role of community culture in addressing elderly issues, and the types of social support available.

### 3.1 Meaning and management of loneliness in the elderly

Participants interpreted loneliness in different ways. Most participants were older women living alone. Referring to de Jong Gierveld et al. (2006), loneliness is a subjective and negative experience, reflecting an individual's cognitive evaluation of their social participation and networks (de Jong Gierveld and van Tilburg, 2010). From the interviews, it was found that many elderly participants did not experience loneliness, while a small number did. Those who did not feel lonely despite living alone viewed the loneliness situationally, recognizing differences between their past and present circumstances and making efforts to understand and accept their situation. Participants who did not feel lonely saw events like the passing of a spouse or children moving out as normal parts of aging, and they accepted their current life while maintaining daily routines. These participants managed their solitude by engaging in various activities or routines and were able to regulate their emotional state, avoiding negative feelings like sadness, anger, frustration, or disappointment. For example, one elderly woman living alone did not feel lonely because she transformed her solitude into a meaningful experience, filling it with beneficial activities, thus nearly eliminating feelings of loneliness. As she expressed in the interview:

“*I don't feel lonely, there is just a difference in the situation. My husband is no longer here, and I live alone. My child lives far away, outside the city for work. I live alone willingly and accept it. I worry about getting sick if I dwell too much on loneliness. When I feel lonely, I usually call old coworkers, friends from religious studies, or my child. Sometimes we video call or chat via WhatsApp. I also reunite with old friends and participate in activities like religious gatherings, elderly exercise, recreation, and feeding the fish in the pond”* (DWI, Women, 76 years).

Situational acceptance and contextual understanding can help avoid loneliness in the elderly. She acknowledges that while she lives alone due to circumstances (her husband's absence and her child living far away), she does not equate this with loneliness. DWI differentiates between physical solitude and emotional loneliness. This distinction is crucial as it reflects her resilience in adapting to changes in her life. A similar experience was shared by LIA, a 67-year-old woman, who understood that her current state of being alone was a situational change. She made efforts to accept her solitude, embracing the remaining years of her life with peace and enjoyment. LIA managed her loneliness by actively engaging in meaningful activities, such as caring for her grandchild who lived nearby. Her participation in various community activities helped her overcome the boredom of staying at home, bringing feelings of happiness and joy through interactions with peers. As she explained in the interview:

“*I am alone, no one else is here. So, I try to understand the situation and accept it. I don't feel affected by being alone like this, I enjoy it. My child lives not far from my house, so sometimes they and my grandchild visit. I often take care of my grandchild and participate in activities in the community here. If I didn't have any activities, I would feel bored, but I join these activities because I enjoy meeting other elderly people here.”* (LIA, Women, 67 years).

LIA's statement illustrates how acceptance plays an important role in managing feelings associated with elderly solitude. Her ability to find enjoyment in solitude while maintaining relationships with family members demonstrates resilience to potential loneliness. Participation in community activities not only combats boredom but also fosters social networks among peers who share similar life experiences. This is important for the emotional health of the elderly. Individuals can positively navigate their later years by embracing their circumstances while remaining connected to family and community, a model for promoting mental health.

This participant gave meaning to her solitude by engaging in meaningful activities, which helped alleviate feelings of loneliness. The subjective interpretation of loneliness depends on individual circumstances and the surrounding environment. These participants demonstrated cognitive, physical, and social capacities, recognizing their situation and using it as a strategy to manage their solitude through various purposeful activities. The involvement of the elderly in community activities aligns with the activity theory proposed by Havighurst et al., which suggests that active participation in activities is key to healthy aging. This theory emphasizes the importance of adapting to changes by replacing lost roles or activities with new alternatives. It posits that maintaining a high level of activity and social interaction is essential for wellbeing and successful aging (Versey, 2015).

YAT, a 76-year-old participant, occasionally experienced loneliness but also moments when she did not feel lonely. She managed her solitude through spirituality, believing that God was always with her and feeling her late husband's presence in the home, despite his long-past death. In addition, activities at home, such as watching television and light interactions with neighbors, helped her overcome loneliness. These strategies provided her with happiness and contentment. As expressed in the interview:

“*I am not afraid of loneliness because God is watching over me, and I feel like my husband is still in this house even though he passed away long ago. When I feel lonely, I usually watch entertaining TV shows, and sometimes I stand outside my house to observe and greet the people around me.”* (YAT, Women, 76 years).

Faith plays an important role in providing comfort and reassurance during times of solitude. Spirituality serves as a protective factor against feelings of isolation (spiritual resilience). The deep emotional connection with her late husband shows that memories can transcend physical absence. This highlights how individuals can maintain bonds with loved ones through memories, which can reduce feelings of loneliness. Coping with loneliness by seeking distraction and pleasure through media consumption and social interaction within the community demonstrates an attempt to connect with others.

The personality of individuals also influences their experience of solitude and loneliness. Individuals with a more introverted nature, who tend to avoid social interaction, have limited social contacts, rarely engage with others, and lack close friendships, may be more prone to loneliness. However, in the case of RUS, an introverted participant, she managed her solitude and loneliness by understanding her situation and engaging in at-home activities such as watching television and YouTube as a form of self-entertainment. For RUS, technology played a vital role in alleviating boredom and loneliness:

“*I rarely go out; I'm a private person and live alone. Yes, I feel lonely, but what can I do since my child lives far away in another city? I usually WhatsApp my child, and when I'm alone like this, watching TV or YouTube is enough to keep me entertained.”* (RUS, Women, 60 years).

Some elderly people use technology such as mobile phones. These devices are used to communicate with relatives and read the news. Some elderly people prefer to look at their mobile phones rather than watch television because they can easily access the news they want. Some elderly people also use telecommunication technology that can give them access to online social relationships. Technology can connect people, but it can also provide superficial interactions that do not fulfill deeper emotional needs. Ss is a 76-year-old senior who uses technology to communicate and find information. She can still communicate online with her old colleagues through her mobile phone. Although online, good and intense social relationships have a good psychological impact on the elderly. “*I prefer to use my mobile phone, look for news through my mobile phone, I can still see the writing on my mobile phone. I still contact my friends through social media”* (SUS, Women, 77 years). The elderly overcome loneliness by managing the situation in a different way. She utilizes telecommunication media to stay connected with others even in her own home. This can improve mood and manage emotions. When the elderly are no longer working, there may be less social contact with coworkers. However, in the current era, the elderly can connect with friends by utilizing telecommunication technology, so physical distance can be overcome.

Acknowledgment of loneliness shows awareness and acceptance of one's emotional state. This acknowledgment is important as it reflects a level of honesty about her experiences. The use of technology for connection shows that technology serves as a bridge to maintain family relationships despite distance. Entertainment choices can accompany moments of solitude. This shows that despite her loneliness, she has found ways to entertain herself through media consumption. Preferring to be alone describes her as introverted, which suggests that she may naturally prefer solitude to social interaction. This self-identification suggests that her feelings of loneliness may not only stem from external circumstances, but also from her personality traits. Positive solitude can be viewed as part of the aging process. Not all the elderly are capable of participating in social activities or community involvement, and we must consider their physical limitations and declining capacities. Elderly individuals with physical limitations or introverted personalities can transform their solitude into a positive experience within their homes. This aligns with the theory of gerotranscendence, which suggests that aging individuals become less preoccupied with social engagement and more selective about their activities, finding value in positive solitude and often experiencing a cosmic connection with the universe (Tornstam, 2005).

Loneliness can negatively impact the mental health of the elderly, and this is exacerbated when unresolved issues or family conflicts are present. Several participants who experienced loneliness had conflicts within their families, stemming from issues such as inheritance disputes, incompatibility between elderly parents and their children, neglect, and other matters. For example, participant EMI felt isolated due to a poor relationship with her family, resulting from an inheritance dispute. Similarly, participant MUR experienced loneliness due to generational conflict and incompatibility with her child. These conflicts disrupted social contact, reduced emotional support, and worsened the psychological state of the elderly. In these cases, the conflict generated feelings of loneliness among the participants. However, the loneliness experienced by these participants was mitigated by community support outside of their family ties. Social engagement and telecommunications technology played a role in reducing loneliness caused by family conflicts.

“*Yes, I feel lonely, but I don't complain. I have a principle that I must stay strong to live my life. I no longer communicate with my family because I am angry about the family inheritance. I live alone, and I've gotten used to it. I need to interact with my neighbors; otherwise, it would be difficult for me. I joined a neighborhood WhatsApp group to stay in touch.”* (EMI, Women, 76 years).

“*Yes, it's lonely being alone at home. My son never interacts with me, even though he lives right next door. I'm upset with him. But the neighbors here are kind, and I often greet them, which makes me happy.”* (MUR, Women, 76 years).

Acknowledgment of loneliness without complaining indicates awareness of her emotional state, she is also proactive in facing challenges. This mindset shows that she values inner strength and self-reliance as important components for navigating loneliness. Unresolved conflicts can cause estrangement and contribute significantly to feelings of loneliness. The significance of community bonds in mitigating feelings of loneliness is acknowledged. The utilization of technological platforms, such as WhatsApp Groups, for community-building purposes is a notable example. The decision to join a WhatsApp group within her neighborhood was a deliberate strategy that not only facilitated her engagement in social interactions but also contributed to the maintenance of relationships within the community, despite physical limitations or a reluctance to engage in face-to-face interactions.

MUR's statement illustrates the complex interplay between familial relationships and community support in shaping one's experience of loneliness. While she grapples with feelings stemming from a lackluster relationship with her son, she also demonstrates resilience by finding joy in neighborly interactions. Her acknowledgment that loneliness exists alongside moments where kindness from others brings happiness reflects an adaptive coping strategy: seeking out positive social connections to counterbalance negative emotions related to family estrangement. Ultimately, this highlights the importance not only of familial ties but also of community bonds in addressing issues related to elderly loneliness.

Conflicts within residential communities, often stemming from differences in perspectives and behaviors, can lead to feelings of social alienation among the elderly. The elderly who do not adhere to social norms may experience exclusion. This was evident in the case of participant TMI, who was socially isolated after failing to comply with the dress code required by the religious gatherings in her community. She faced sanctions, which deepened her sense of loneliness.

“*I joined the religious gathering, and we were required to wear a uniform. I didn't follow that rule, and as a result, I was penalized by not being allowed to participate anymore. I don't want to create enemies in this community.”* (TMI, Women, 63 years).

TMI's statement illustrates the tension between individual identity and communal expectations within religious or social groups. While she seeks connection through participation in religious gatherings, adherence to specific rules becomes a barrier. Impact of social norms: The enforcement of uniformity within communities can lead individuals like TMI to feel alienated if they do not conform, even if their intentions are rooted in personal choice rather than defiance. The importance she places on maintaining positive relationships within her community despite facing exclusion due to non-compliance. TMI's experience reflects broader themes related to conformity, belongingness, individuality vs. collective identity, and the complexities involved in navigating social interactions within structured communities.

Physical limitations further exacerbated loneliness. Participant ASI, who suffered from a degenerative illness, was unable to participate in social activities due to her health condition, which hindered her from engaging with the community. Physical restrictions can trap elderly individuals within their homes, preventing social interactions. ASI managed her situation by engaging in solitary activities at home, focusing on spiritual practices:

“*I can't (engage in outdoor activities), and I'm afraid if I fall ill, there will be no one to take care of me. When I feel lonely, I do activities at home (such as praying).”* (ASI, Women, 66 years).

ASI's statement reflects the interplay between health concerns and emotional wellbeing. Her fear of illness underscores the importance she places on having reliable support during vulnerable times; this concern is compounded by her inability to engage in outdoor activities due to potential health issues or mobility limitations. Her reliance on prayer as a coping mechanism illustrates how spirituality can serve as both an emotional refuge and a source of strength when faced with loneliness. While she acknowledges feelings of loneliness, ASI demonstrates resilience by finding ways to maintain some level of engagement with herself through prayer despite external circumstances limiting broader social interactions. ASI's experience highlights the challenges faced by elderly individuals who may struggle with both physical limitations and feelings of loneliness while also seeking comfort through personal beliefs or practices within their homes.

These cases highlight the subjective nature of loneliness among participants, with each individual responding and managing their circumstances in different ways. Their unique approaches reflect their personal strengths, capacities, and strategies for mitigating loneliness. Despite these variations, there are similarities in how participants managed their situations, particularly in the form of local community support. The following section discusses how community involvement plays a crucial role in helping elderly cope with loneliness and social isolation.

### 3.2 Collectivism in the Simomulyo Baru community

In societies with individualistic cultures, loneliness is more prevalent due to lower social contact and social cohesion (Barreto et al., 2021; Chow et al., 2021; Heu et al., 2021). Individualistic cultures are often associated with urban populations (gesellschaft), where there is high mobility and cultural acculturation. In such societies, individuals tend to spend significant time in economic activities, leading to greater diffusion of culture and social practices that influence local culture.

Simomulyo Baru is a neighborhood located in the heart of Surabaya, surrounded by office buildings, economic centers, dense housing, and a diverse population. The acculturation of Javanese and Chinese cultures is evident, alongside the presence of migrants from outside the city. Despite the urban environment, the research revealed that social cohesion and solidarity still exist within the community of Simomulyo Baru. The sense of togetherness, or guyub, altruism, respect for the elderly, and harmony are reflected in this community. Guyub, similar to the characteristics of gemeinschaft, is formed through the social practices of the actors within the community. Each actor follows shared values and norms, which then become ingrained in the culture. According to a study by Schirmer and Michailakis (2015), one of the causes of loneliness in contemporary society is the loss of *gemeinschaft* (community). *Gemeinschaft* refers to a small community characterized by high integration, social cohesion, solidarity, closeness, and familiarity.

Guyub (togetherness) and rukun (harmony) are values deeply embedded in Javanese society, Indonesia. A culture of guyub fosters a sense of care (altruism), mutual respect, and harmony. In this study, the guyub culture in the Simomulyo Baru community has been shown to help elderly individuals who live alone cope with loneliness. Simomulyo Baru is an urban community with significant cultural acculturation, where many migrant groups have settled. The values of togetherness, care, and respect for the elderly within this community contribute to reducing loneliness among the elderly, as they feel noticed, happy, and comfortable within their community. The community serves as a tool for social empowerment for elderly individuals living alone and experiencing loneliness. The residential community (neighborhood) is the closest community to the elderly, with a stronger sense of togetherness compared to family members who live far away. This solidarity is evident in social practices such as conversing, sharing meals, visiting when ill, and going to religious gatherings. “*Everyone here is kind, they often check on me and give me food. People here are guyub—it's like my own family.”* (SUS, Women, 77 years old).

*Another participant added: “I gather with friends and neighbors, I've lived here a long time so it's comfortable. I make sure there's no misunderstanding with neighbors to maintain harmony. When I'm sick, they visit me, they care about me. If I don't leave the house for a few days, they come by, remind me not to lock the house, and I appreciate them*. I live alone and they watch out for me, so I feel I must engage with the community.” (EMI, Women, 76 years old).

The statements reflect a profound sense of belonging among elderly individuals within their community. Their experiences illustrate how strong social networks can significantly alleviate feelings of loneliness. The cultural values associated with guyub foster an environment where mutual aid is commonplace, enhancing emotional wellbeing. Their narratives highlight not only individual resilience but also collective strength derived from supportive relationships within their neighborhood. These insights underscore the critical role that community dynamics play in promoting mental health among older adults by providing them with companionship, care during illness, and opportunities for social interaction.

Social practices within the community create a social reality, establishing patterns of interaction that become ingrained in the culture. Collectivism here emerges from the positive social practices within the community and serves as an intervention tool for elderly individuals experiencing solitude and loneliness. Communities also serve as a medium for social cohesion, with many activities organized by the community that promote social interaction and integration with rules and norms that bind and become traditions. Religious and community social activities encourage elderly participation, which ultimately reduces feelings of loneliness and fosters a sense of togetherness. “*Neighbors are our closest family. We must engage with the community; socializing is life. There are many ways to gather with those around us, such as joining a social club.”* (DWI, Women, 76 years old). The statement from DWI, emphasizes the importance of community and social engagement in her life. She is reframing relationships. Neighbors as family, which suggests that she views her neighbors not just as acquaintances but as integral parts of her support system. This perspective highlights the significance of community ties, especially for elderly individuals who may have lost family members or live far from them. Value of Social Engagement: Socializing is Life. The essential role that social interactions play in maintaining emotional wellbeing and overall quality of life. human connections are vital for mental health, particularly in later years. Her emphasis on the necessity to engage with the community indicates a proactive approach to combating loneliness. She recognizes that active participation can lead to fulfilling relationships and a sense of belonging. The mention of joining a social club illustrates practical ways individuals can foster connections with others around them. It suggests an openness to new experiences and interactions, reinforcing the idea that there are various avenues available for building relationships within one's community.

“*There's a religious gathering here, and after the activity, we eat together. Even though there are many migrant groups, we maintain harmony. Life is about community, not arguing with neighbors—neighbors are family. We have traditions like bringing sugar, oil, and onions for community events. I've lived here for a long time, and my neighbors feel like family. For the elderly, what's most important is talking and communicating. For example, I go to the mosque with my neighbors for prayers, and we see each other during communal prayers.”* (LIA, Women, 67 years old).

Religious gatherings highlight the importance of faith as a unifying force within the community. These gatherings serve not only spiritual purposes but also act as social events that strengthen bonds among members. The practice of eating together after activities emphasizes communal living and shared experiences. This ritual fosters connection, belonging, and mutual support among participants. Cultural Diversity and Harmony of Migrant Groups suggests a diverse community where individuals from different backgrounds coexist. They maintain harmony, indicating an effort to foster inclusivity and understanding among various cultural groups. Cultural traditions as social glue. Mentioning traditions in community events illustrates how shared customs contribute to social cohesion. These practices create opportunities for interaction while reinforcing cultural identity within the community.

Culture plays an important role in shaping the experience of loneliness among individuals. Individuals from collectivist cultures, where there is a strong emphasis on group connection and harmony, experience loneliness differently (Hämmig, 2019). Cultures that value life organization and place strong emphasis on family and community support can help reduce feelings of loneliness in the elderly. The forms of culture present within a community influence the loneliness experienced by the elderly. Some elderly participants do not feel lonely due to the cohesive cultural community, adherence to religious values, and social norms present in society. But there was one elderly person who felt alienated due to strict social norms. This strictness of social norms serves to regulate and shape collectivism. *Gemeinschaft* (community) exists in cultural-type societies. Cultural aspects become an integral part of the subjectivity of perception and consciousness (Dmitrievna et al., 2014).

### 3.3 Social support for the elderly

Sources of support for the elderly, especially those who live alone, are crucial to reducing feelings of loneliness. Support can come from family, friends, communities, and others. Among the elderly, feelings of loneliness can be mitigated by the presence of children and grandchildren. Spending time with family members provides emotional support, fostering communication and interaction with them. Family, being the closest and most accessible space for the elderly, can offer significant support.

Social changes have eroded the roles of families, especially in urban areas. There has been a shift in the roles within the family, including the elderly, children, and grandchildren. Elderly people no longer receive full care from their working children. For example, working daughters may no longer provide full-time care for their elderly parents. Another role shift is that the elderly may now care for young grandchildren. This shift in roles impacts the elderly, both positively and negatively. On the positive side, the relationship between elderly individuals and their grandchildren allows them to internalize cultural values and societal norms through caregiving. On the negative side, some elderly may experience fatigue due to their physical limitations.

However, emotional support from grandchildren can alleviate feelings of loneliness. Grandchildren, especially those still young, offer emotional support (entertainment and happiness) to the elderly. The term “momong cucu” (grandchild care) represents a shift in the roles of the elderly in contemporary society. Taking care of and raising grandchildren has become a common shift in contemporary communities. Grandchildren are a source of support for elderly people experiencing loneliness. Their presence in the lives of the elderly provides emotional support and creates new roles for them. The presence of family members gives elderly people a sense of purpose and reduces feelings of loneliness, thus improving their quality of life.

“*Every day my grandchild comes to my house after school from noon until evening. After his father comes home from work, he returns to his own home. I take care of my grandchild, such as making him food and taking him to school”* (LIA, Women, 67 years).

LIA describes her daily routine involving caring for her grandchild. This indicates that she has an active role in the family structure, contributing significantly to the child's wellbeing and development. The regular presence of her grandchild after school suggests a strong intergenerational bond. This relationship likely provides emotional fulfillment for her, as caring for grandchildren can enhance feelings of purpose and connection. The structured nature of LIA's day (daily routine) provides stability not only for the child but also for herself. Routines can be comforting, especially in later life when changes may lead to feelings of uncertainty or loneliness. Social Interaction with family by taking care of her grandchild maintains social connections within the family unit. This interaction is crucial as it helps mitigate feelings of loneliness that some elderly individuals may experience.

The demands of modern society, where family members work outside the home, often lead the elderly to take on the role of caring for their grandchildren. The elderly also influence their grandchildren, particularly in terms of their social and cultural development. Role changes among the elderly impact institutional support, attitudes, identities, and intergenerational interactions (Tan Jun Hao and Ng, 2010). Relationships with family members become crucial for the mental health of the elderly and help reduce feelings of loneliness. The role of the elderly is central to intergenerational solidarity, and taking care of grandchildren has been identified as an essential form of multigenerational family emotional support. With increasing life expectancy, elderly individuals are spending more time with their grandchildren than before (Tsai and Chen, 2023).

Another source of social support for the elderly includes close kin, such as siblings. For elderly individuals who are distant from their children, the nuclear family may not be relied upon for care. Siblings can provide assistance when an elderly person living alone requires help. ASI statement underscores the complexities faced by elderly individuals living alone, particularly those distanced from their children. It illustrates a duality, while there is a desire for independence and self-sufficiency, there is also an acknowledgment that social connections especially with siblings that can play a vital role in providing necessary support. The importance of maintaining familial ties beyond immediate nuclear families. The need for community or sibling networks to fill gaps left by distant children. A recognition that independence does not preclude the necessity or benefit of social connections; rather, it can coexist with them. Overall, this narrative reflects resilience in facing solitude and an understanding that relationships with close kin remain essential even when traditional family structures (like those involving children) are less accessible.

“*I live alone at home. No one is taking care of me. I must be independent, not depend on anyone. My children are far away in another city. So, my younger sibling comes when I need help”* (ASI, Women, 66 years).

Support from close family members offers emotional support for the elderly. According to Liu, S., affection is one form of emotional support that can improve the elderly's mental health. Nowadays, many children are no longer able to fulfill caregiving responsibilities for their elderly parents due to economic demands. Elderly people take on new roles, such as caregiving for grandchildren. This role brings a sense of happiness to the elderly, where grandchildren provide positive moods, comfort, and joy, particularly for those who live alone. For the elderly, when they are caught in situations of solitude and loneliness, the presence of family members like grandchildren and the caregiving role can improve their mental health.

The lost role of children in directly providing services to the elderly can be replaced through indirect forms of emotional support, such as expressing love, concern, and listening to the elderly's complaints, even through telecommunications apps like WhatsApp or video calls. As experienced by participant DWI, despite living alone, she still communicates with her children and grandchildren through WhatsApp, providing emotional support. “*Yes, sometimes I make video calls, sometimes I WhatsApp with my children and grandchildren. They are far away in other cities, they ask how I am doing”* (DWI, Women, 76 years).

In Simomulyo Baru, communities play a secondary role as the backdrop for the lives of individuals and groups. The social capital developed within communities has significant implications for the health and wellbeing of the elderly. Social groups impact both the physical and mental health of the elderly. Residential communities (neighbors) are among the most influential social groups because neighbors are the people closest to the home. The variety of social groups and communities allows the elderly to engage in social activities within easily accessible distances. Local communities provide social cohesion for their residents, offering meaning and purpose in life beyond mere social interactions. Neighborhood and community social support strengthen the elderly and help reduce loneliness (McNamara et al., 2021).

Communities serve as secondary sources of social support for the elderly. Identifying community and social support can reduce loneliness, and communities act as intervention tools. The role of communities shapes the social life of their inhabitants, influencing social practices and societal realities. In this study, the non-kin primary groups include residential communities (neighbors), social groups like religious groups and elderly social organizations. The community can provide companionship support for the elderly. One of the main needs of the elderly in contemporary society is companionship. Companionship support is essential for elderly individuals living alone to combat loneliness. This support positively impacts the elderly by providing opportunities to interact beyond family members.

“*I was invited to my children's house, but I didn't want to go. Their housing area is very closed, and there are no elderly friends there. Here, I have many elderly friends. When you get old, the most important thing is talking, communication. If you don't have anyone, you rely on neighbors”* (LIA, Women, 67 years).

The emphasis on having many elderly friends suggests that peer relationships provide comfort and companionship that help mitigate feelings of loneliness. This statement highlights how vital it is for elderly individuals to maintain active social lives within their communities rather than relying solely on family interactions, especially when geographical or situational factors limit those familial connections. Companionship support is crucial for elderly individuals to combat loneliness. This suggests that social interactions with peers can significantly enhance their emotional wellbeing. LIA underscores that friendships with peers can be more fulfilling than family interactions in certain contexts, especially when those family members do not provide opportunities for social engagement.

Gathering with peers and engaging in activities gives elderly people joy, helps alleviate boredom, and improves mental health. Residential groups can strengthen elderly individuals who feel lonely. “*My neighbors are all harmonious, they care about me. We often gather for prayers and elderly exercise. I'm happy to have so many friends”* (MUR, Women, 76 years). MUR's words illustrate how active participation in community life through prayer gatherings and exercise can lead to increased happiness, reduced feelings of loneliness, and improved overall mental health among elderly individuals. Her appreciation for her neighbors indicates that strong interpersonal relationships play a crucial role in enhancing the quality of life during later years. The emphasis on harmony within her community further underscores the importance of supportive environments in fostering resilience against loneliness.

Saving clubs and religious gatherings are also activities frequently participated in by the elderly. They join these activities to increase engagement and social interaction. These activities often take place once a week, like elderly exercise, or once a month, like community savings groups. Religious activities happen daily at specific times. “*I attend the prayer group, savings club, and PKK to stay busy. I do exercises once a week, savings clubs once a month, and go to the mosque for prayers every day at dawn, dusk, and night”* (NAR, Women, 81 years). The excerpt illustrates how participation in various clubs and gatherings serves multiple purposes: it combats loneliness by fostering social connections, promotes physical health through exercise, and provides structure to daily life through regular engagement with peers. For many elderly, these activities are not just pastimes, they are essential components that contribute to overall wellbeing (emotionally, socially, and physically). Organized group activities, such as prayer groups or savings clubs, play a crucial role in enhancing social interaction among the elderly. Regular participation helps maintain mental stimulation while also addressing feelings of isolation. These engagements reflect both personal agency (the desire to stay busy) and community involvement (connecting with others), ultimately enriching their quality of life.

According to several participants, gathering with the local community is a pleasant activity that boosts immunity for the elderly. These activities are often held close to their homes, making them easily accessible. The elderly activity group is called Paguyuban Werdho Tomo, or the Elderly Association. This space allows the elderly to engage in peer communication, physical fitness, health checks, charity activities, and occasionally recreational activities. It facilitates interactions, strengthens physical, psychological, and mental health, and fosters a sense of community unity.

“*I want activities so I don't become senile. The elderly's dependence needs to be helped, and more attention should be given to the elderly. Here, there's an elderly group (residential community), called Paguyuban Werdho Tomo. The location is easily accessible for the elderly, with activities like exercise and sometimes recreation”* (SUS, Women, 77 years).

The statement provides insights into the benefits of community engagement for elderly individuals, particularly through organized groups. The statement emphasizes how participation in community gatherings significantly benefits elderly individuals by promoting both physical health (through fitness activities) and mental wellbeing (through social interactions). The existence of accessible groups like Paguyuban Werdho Tomo plays a vital role in creating supportive environments where seniors can thrive socially, physically, and emotionally. Overall, this reflects an understanding that holistic approaches, combining fitness, communication, charity work, and recreation are essential for improving the lives of older adults within their communities.

Community engagement as a health booster. Social interactions are not only enjoyable but also contribute positively to the wellbeing of elderly individuals (pleasant activities). This aligns with research indicating that social engagement can enhance mental and physical health. Paguyuban Werdho Tomo (Elderly Association) as a structured support System. Establishing an organized group like Paguyuban Werdho Tomo provides a structured environment where elderly individuals can engage in various activities tailored to their needs. Peer communication facilitates social interaction, reducing feelings of loneliness. Regular exercise contributes to better physical health and mobility. Health checks promote preventive care among the elderly. Charity activities foster a sense of purpose and community involvement. Recreational activities provide opportunities for relaxation and enjoyment, further enhancing quality of life. Paguyuban Werdho Tomo, according to a community leader and founder of the elderly association, provides a space for elderly individuals to engage in social interactions and strengthen social cohesion, thus contributing to a reduction in feelings of loneliness among the elderly. In this social space, the elderly can participate in various activities such as talking with fellow elderly members, visiting those who are sick, attending elderly activities like exercise and walking, celebrating national holidays, and engaging in recreational activities for seniors. As quoted from an interview with SUG:

“*Elderly people cannot engage in many activities. They are over 60, have grandchildren. Werdho Tomo emphasizes the elderly, prioritizing their well-being. The aim is to boost their immunity. Initially, it was socialized through a WhatsApp group. It's not easy for the elderly to attend, it requires an approach. We care for each other; if someone is sick, we visit. Over time, elderly people who didn't participate started joining, and many became interested. There are activities like walking, prayers, free health checks, and recreation. Elderly people who often gather with peers will feel happy. If they have physical limitations, just attending community activities and talking with other seniors makes them happy, and it boosts their immunity. They also get bored looking after their grandchildren.”* (SUG, Women, 78 years).

Community support is crucial for elderly individuals living alone, acting as an intervention due to the impacts of social changes. Community support can replace the physical absence of family support. The existence of Paguyuban Werdho Tomo provides opportunities for social connections, strengthens social cohesion with the local community, and positively impacts the physical and mental health of the elderly. Social reality is shaped by social practices, and the role of the community is a key determinant of this reality. Several elderly participants in this space tend to not feel lonely. Social relationships within the community provide positive support for the health and wellbeing of the elderly.

Another issue for the elderly is financial problems, often due to retirement or unemployment. Elderly people need financial support. Material support, in the form of money and facilities, provided by family, especially children, is very important for those living alone. Even if not living with the elderly, children offer material support as a form of respect and indirect care, as experienced by participant ASI: “*My children work out of town. I live alone. They give me money for my needs here because I don't work”* (ASI, Women, 66 years). This statement reflects a critical aspect of aging: the intersection between financial stability and social relationships within families. It underscores how essential it is for families to provide both emotional and material support to ensure that elderly individuals can maintain a decent quality of life despite potential isolation or economic hardship caused by retirement or unemployment. As they age, their income sources may diminish, leading to financial insecurity. Importance of material support for the elderly. The statement stresses that elderly people require financial support, particularly those living alone. This indicates that without adequate resources, their quality of life may be compromised. The text points out that material support, both material and in terms of facilities, is crucial for elderly individuals who are isolated or living independently. It highlights the role children play in providing this support as a form of respect and care (the role of family in providing material support). Even if children do not live with their parents, their provision of material assistance is seen as an indirect form of care. This suggests that emotional connections remain strong despite physical distance.

From observations, there appears to be a lack of instrumental support, aside from family, and informational support was observed through the use of information technology. The elderly have utilized the internet to access information about elderly life, health maintenance strategies, and more. Social support, both internal and external to the elderly, positively contributes to their mental health. Elderly people are happier when they receive affection, engage in relationships, share with others, and offer mutual support, even if they live alone.

Collectivist culture and various forms of social support help reduce loneliness among the elderly due to social changes. Culture and social support provide social and mental reinforcement through connections, social cohesion, altruism, and respect for the elderly. This social reinforcement has a significant impact on the mental health and wellbeing of elderly individuals living alone at home. The role of family can be replaced by community support, where neighbors take care of the elderly in their environment. Social practices within the community reflect the social reality of the participants of this research, where most elderly individuals living alone do not experience loneliness. Loneliness in the elderly is more often caused by generational conflicts and differences. However, the community's role in culture and social support, especially their care for the elderly, can minimize loneliness resulting from such issues.

## 4 Discussion

### 4.1 Loneliness and changing the situations

Loneliness is a significant mental health risk for the elderly, yet many elderly lack awareness of the importance of managing solitary conditions, often exacerbating their situation. However, findings indicate that elderly female participants experiencing solitude generally possess sufficient ability to transform this situation into meaningful activities and routines. They view loneliness as an acceptable part of aging, understanding that growing older often involves family members becoming less present due to social and familial shifts. Social changes have left the elderly increasingly vulnerable (Langmann, 2023) due to the reduction or loss of nearby social support and family-based care. This vulnerability is compounded by weakening intergenerational relationships, increased mobility, and individualistic societies (Fakoya et al., 2020).

This research is devoted to elderly women because in that location, there are many elderly people who live alone. As is known, the assumption is that elderly people who live alone at home will be more vulnerable to loneliness. But the research findings reveal an inverse reality, that some elderly women do not experience feelings of loneliness. This study shows that many older women who live alone do not necessarily experience feelings of loneliness. There are several factors that influence this. Elderly women often have the ability to adapt to situations of loneliness. They can find ways to turn these circumstances into more positive and productive experiences (adaptive ability). Involvement in community activities, such as social activities or hobbies, gives them the opportunity to interact with others and create meaning from time spent alone (Engagement in meaningful activities). Many elderly women demonstrate a sense of acceptance of their circumstances, accepting the realities of life that may involve being alone without feeling down. This resilience helps them deal with the emotional challenges that come with loneliness (acceptance and resilience). Elderly women have the capacity to register the stimuli around them, be it social interactions, natural beauty, or community activities, and transform them into meaningful situations through reflection and active action (environmental observation capability).

As a subjective evaluation of individual circumstances (de Jong Gierveld and van Tilburg, 2010), loneliness is experienced differently by each participant. For some elderly individuals, this phase of life is acceptable, with loneliness perceived and managed subjectively, informed by their knowledge and evaluation of their solitary experiences. Those with sufficient awareness and evaluative capacity can transform potentially isolating situations into meaningful ones through activities within the home and social engagement. While elderly individuals living alone may appear isolated, research shows they are not necessarily alienated or lonely. Among eleven elderly female participants living alone, three reported loneliness due to conflict, illustrating that loneliness here is not linked to the quantity of social connections but rather the quality. Family conflict can reduce relationships and emotional support. The presence of a family with emotional support can reduce loneliness in the elderly, therefor conflict should be avoided. The perception and experience of loneliness hinge on personal evaluation and the capacity to create meaningful situations and on processes of social exclusion.

A willingness to transform solitude into beneficial, meaningful experiences reflects an individual's capacity to address personal challenges. In addition to physical capacity decline, aging adults face diminishing social capacities, potentially worsening isolation and loneliness. Strengthening social capacity can mitigate loneliness, for instance, through community engagement, which helps build social resources. Cultural expectations may also drive individuals to develop social capacities, ultimately reducing loneliness among the elderly.

### 4.2 Culture and social reality

Social practices within the community create a social reality in Simomulyo Baru, wherein social cohesion and integration help prevent loneliness among elderly individuals, even those living alone. Over time, these community practices become embedded as cultural norms, reflecting values that emphasize respect for the elderly, togetherness, familial bonds, and care, which guide individual behaviors. Actions grounded in these values and norms foster social harmony and order. Communities facilitate a supportive environment for the elderly, provide social engagement activities, and improve the quality of life for the elderly through social support, thereby reducing loneliness. In a collectivist culture, known locally as “guyub,” communal values shape individual behavior to promote solidarity and social integration.

In collectivism, each member is expected to follow the prevailing norms. This is intended to maintain cohesion and harmonization. Therefore, it will strengthen social cohesion. Collectivism provides a shared identity, order, and collective action that will create social cohesion. Meanwhile, members who do not act according to the norms or rules are given violations as social sanctions; this is social control to strengthen group identity, conformity, integrity, and harmony. Those who deviate will be sanctioned or ostracized. Inclusion and exclusion are social adaptation mechanisms to maintain harmony and group identity. The elderly who experience exclusion, like TMI, come from the lower-middle class. She is not unwilling to follow the norms in the group, but at a certain time, she is wrong in following the rules and then experiences ostracization as a result of her actions. The elderly experience exclusion for not following norms due to cognitive and economic limitations. In collectivist societies characterized by high social cohesion, strict adherence to social norms is expected, and individuals who deviate from or fail to conform to these norms risk social ostracism.

Research by Barreto et al. (2021) suggests that social and cultural factors influence individuals' subjective experiences of loneliness, with personal backgrounds further shaping this meaning (Chow et al., 2021). For participants, loneliness is interpreted through the community's values and norms. The principles of togetherness, familial unity, tolerance, and care—central to the collectivist “guyub” culture—manifest in social interactions and community activities, which are seen as social responsibilities for all members. Participants engage in community spaces to foster shared connections and support, with community rules and activities aimed at strengthening unity and fostering attitudes of care, respect, and social harmony.

Subjective perceptions of loneliness among participants are largely mitigated by the community's collectivist culture, values, norms, and activities. Even elderly women living alone find substantial support within the community, benefiting from the “guyub” culture that assists them in various aspects of life. Community spaces and activities encourage social involvement, promoting social cohesion and serving as a source of support for elderly individuals who have lost physical family presence due to social changes. These social practices foster a culture that shapes social reality in Simomulyo Baru, where elderly women living alone may not necessarily experience loneliness.

Strong Family Bonds: In collectivist cultures, a close-knit structure can reduce feelings of loneliness as they have regular interactions that provide emotional support. Collectivist societies encourage participation in community activities and gatherings. This engagement fosters social connections among elderly individuals, helping to alleviate feelings of loneliness. Aging is viewed positively; elders are respected and seen as bearers of wisdom. This respect can enhance their self-esteem and mitigate feelings of loneliness.

### 4.3 Family and community social support

Limited social relationships and support heighten the vulnerability of elderly individuals, particularly those living alone. While some elderly female participants live independently, others occasionally receive family visits. Shifts in family roles have led many elderly to adapt to solitary living, as families often face constraints in providing direct care. Physical and geographical separation from family challenges participants to independently manage solitude and loneliness. However, physical absence of family does not necessarily equate to a lack of social support. Many elderly participants effectively manage solitude and maintain family support through telecommunications technology. Melchiorre et al. (2024) indicate that when social support is limited, technology can help the elderly stay connected to family, friends, and the community. Some participants use social media platforms, like WhatsApp, for virtual family interactions, including calls and video chats. While irreplaceable, family remains the primary provider of emotional and social reinforcement for the elderly.

Social support encompasses resources provided by others for assistance, comfort, and reassurance (Santos and Hamdan, 2024). Support functions provided to the elderly come from family, friends, neighbors, professionals, and social organizations (Liu, 2019). Research identifies primary sources of support as close kin (e.g., children, grandchildren, siblings) and secondary, non-kin primary groups (e.g., friends, neighbors, community members). However, findings show an absence of extended kin and secondary group support, both increasingly critical in modern society for addressing elder care issues preventively.

Regular interaction with friends or family provides emotional comfort for older adults facing loneliness. Knowing that there are people who care about them can significantly improve their mental wellbeing. Help with daily tasks from social networks reduces the stress associated with aging alone while fostering a sense of connectedness. Social groups (e.g., clubs or religious gatherings) offer structured opportunities for interaction among peers that help combat isolation through shared experiences. A strong social support system acts as a buffer against life stressors such as health problems or loss; this buffering effect contributes to the perceived level of loneliness.

The study notes three main support functions for the elderly: material support, emotional support, and companionship support, yet finds no instrumental or informational support. This gap is tied to the absence of a secondary group, where instrumental support could aid preventive and curative elderly care efforts. Professionals such as social workers, doctors, and psychologists can serve as key sources of informational support. A preventive approach to elderly care should be expanded to reduce social issues in aging populations, promoting healthy, active, and productive aging. Given the growing aging demographic, establishing secondary groups to provide instrumental support is crucial. Expanding informational support offers knowledge and skills development to enhance the elderly's capacity.

## 5 Conclusion

Loneliness was interpreted uniquely by each participant, reflecting its subjective nature. Therefore, it is inappropriate to assume that living alone always equals loneliness and therefore vulnerability. Moreover, accurately understanding loneliness requires a comprehensive understanding of the individual's background, including their personal history, family dynamics, community context, challenges and needs. Elderly manage feelings of loneliness by identifying and leveraging available sources of social support in their environment. These social supports serve as psychological and social resources that can strengthen their mental wellbeing and fill making their solitude and loneliness meaningful.

Loneliness in the elderly is the subjectivity of the elderly and becomes a limitation in this study, it cannot be generalized at the population level. Elderly subjectivity regarding loneliness is shaped by the local social and cultural environment, as expressed by Barreto et al. (2021). Although many elderly people live alone, “guyub” can provide social glue so that social relationships are created through rules or norms. As stated by Heu et al. (2021) culture shapes relationships and maintains social cohesion in society. Elderly with lower-middle and upper-middle social classes who are surrounded by a culture of collectivism generally get social support from family and community, so that they can reduce loneliness. In the current era, many elderly people are left behind by family members for certain reasons, but they are able to adapt and accept the situation, which is transformed into meaningful activities with the community in a culture of guyub. Social support from family and community is the closest intervention strategy for the elderly to reduce loneliness. This also confirms the findings that social support can overcome the challenges of adjustment in the elderly. From the results of the study, the social support available to elderly participants was more dominant in material support, emotional support, and companionship support. Meanwhile, informational and instrumental support are not yet available in research locations.

The role of culture, values, and norms binds individuals to behave according to societal rules, which can create social cohesion. The culture of collectivism (Guyub) also fosters social support for the elderly. The social context embodies social integration through a set of rules for social relations in society. Social context can also influence the elderly's subjectivity about loneliness. Social context can also influence the elderly's subjectivity about loneliness, the elderly will identify their environment The elderly will recognize their environment and then interpret their loneliness. Regardless of the differences in the social class of the elderly, they can potentially experience loneliness due to various factors. Although social class can shape the views and actions regarding loneliness in the elderly, this study show that any social class can potentially experience loneliness. Different types of social support should be considered as interventions for elderly individuals experiencing loneliness. Social support creates harmonious social relationships. The elderly's involvement in their community is a way to achieve companionship support, although it also engenders exclusionary processes. Policymakers and social practitioners need to consider various types of social support, such as instrumental and informational support, as a comprehensive strategy to address issues faced by the elderly. This includes strengthening the roles of family and community to combat loneliness among the elderly.

## Data Availability

The original contributions presented in the study are included in the article/supplementary material, further inquiries can be directed to the corresponding author.
